# Explaining demand patterns during COVID-19 using opportunistic data: a case study of the city of Munich

**DOI:** 10.1186/s12544-021-00485-3

**Published:** 2021-04-12

**Authors:** Vishal Mahajan, Guido Cantelmo, Constantinos Antoniou

**Affiliations:** Chair of Transportation Systems Engineering, Department of Civil, Geo and Environmental Engineering, Technical University of Munich, Arcisstrasse 21, Munich, 80333 Germany

**Keywords:** COVID-19, Demand patterns, POIs, Spatial-temporal, Crowdsensed data, Machine learning

## Abstract

**Background:**

The COVID-19 pandemic is a new phenomenon and has affected the population’s lifestyle in many ways, such as panic buying (the so-called “hamster shopping”), adoption of home-office, and decline in retail shopping. For transportation planners and operators, it is interesting to analyze the spatial factors’ role in the demand patterns at a POI (Point of Interest) during the COVID-19 lockdown viz-a-viz before lockdown.

**Data and Methods:**

This study illustrates a use-case of the POI visitation rate or popularity data and other publicly available data to analyze demand patterns and spatial factors during a highly dynamic and disruptive event like COVID-19. We develop regression models to analyze the correlation of the spatial and non-spatial attributes with the POI popularity before and during COVID-19 lockdown in Munich by using lockdown (treatment) as a dummy variable, with main and interaction effects.

**Results:**

In our case-study for Munich, we find consistent behavior of features like stop distance and day-of-the-week in explaining the popularity. The parking area is found to be correlated only in the non-linear models. Interactions of lockdown with POI type, stop-distance, and day-of-the-week are found to be strongly significant. The results might not be transferable to other cities due to the presence of different city-specific factors.

**Conclusion:**

The findings from our case-study provide evidence of the impact of the restrictions on POIs and show the significant correlation of POI-type and stop distance with POI popularity. These results suggest local and temporal variability in the impact due to the restrictions, which can impact how cities adapt their transport services to the distinct demand and resulting mobility patterns during future disruptive events.

## Introduction

People undertake different activities during the day to satisfy their needs and wants. The performance of such activities depends on various population and environmental factors. The interaction of land-use and transport systems in activity generation has also been widely researched and modeled [1]. Places well connected with transport systems and dense neighborhoods will generate more trips than less connected and sparse neighborhoods. The interaction between land-use and transport and individual constraints is captured by the concept of accessibility [2, 3]. Apart from the above “structural” factors, planned or unplanned special events [4], weather conditions [5, 6], and others, can also influence in the short-term, where, when, and how people move. Cities strive to plan, design, and operate their transportation systems based on the forecasted demand, derived out of the activities due to these factors.

In case of disruptive and highly dynamic events, such as natural or human-made hazards, people tend to adapt their short-term [7] and long-term mobility behavior [8, 9] to the prevailing conditions. For example, travelers might be reluctant to enter the underground metro after an earthquake or go near the sea-coast in case of a cyclone or tsunami warning. COVID-19, one of the most severe pandemics in the last 100 years, has affected almost the entire world in unprecedented ways. To control COVID-19 transmission, guidelines such as social distancing, masks, and movement restrictions were recommended or enforced. In response to these measures, people not only reduce their mobility [10], but they also adapt their travel patterns in order to limit their exposure by avoiding places with a high number of cases [11]. The anticipation or announcement of movement restrictions due to COVID-19 causes specific changes in people’s lifestyles, routines, and consumption patterns, such as panic buying during the early pandemic or lockdown stage [12], working from home, or a decline in non-essential retail consumption [13]. With people spending on average, around 40% less, these new trends also risk generating an economic slowdown that could last for a long time [13]. Such changes in the behavior and attitudes, if significant, can reveal a pattern exhibited through changes in the number, types, and spatio-temporal extent of the activities. For example, crowding at some locations or imbalanced use of transport facilities, like roads and transport modes, can be observed. Planners must understand these behavioral changes and, more importantly, the spatio-temporal patterns of the population’s activities for an effective response. The scale and speed of these changes have left cities, transport operators, and research communities with several unanswered questions on how to respond so that a basic service level is efficiently maintained.

**Fig. 1:**
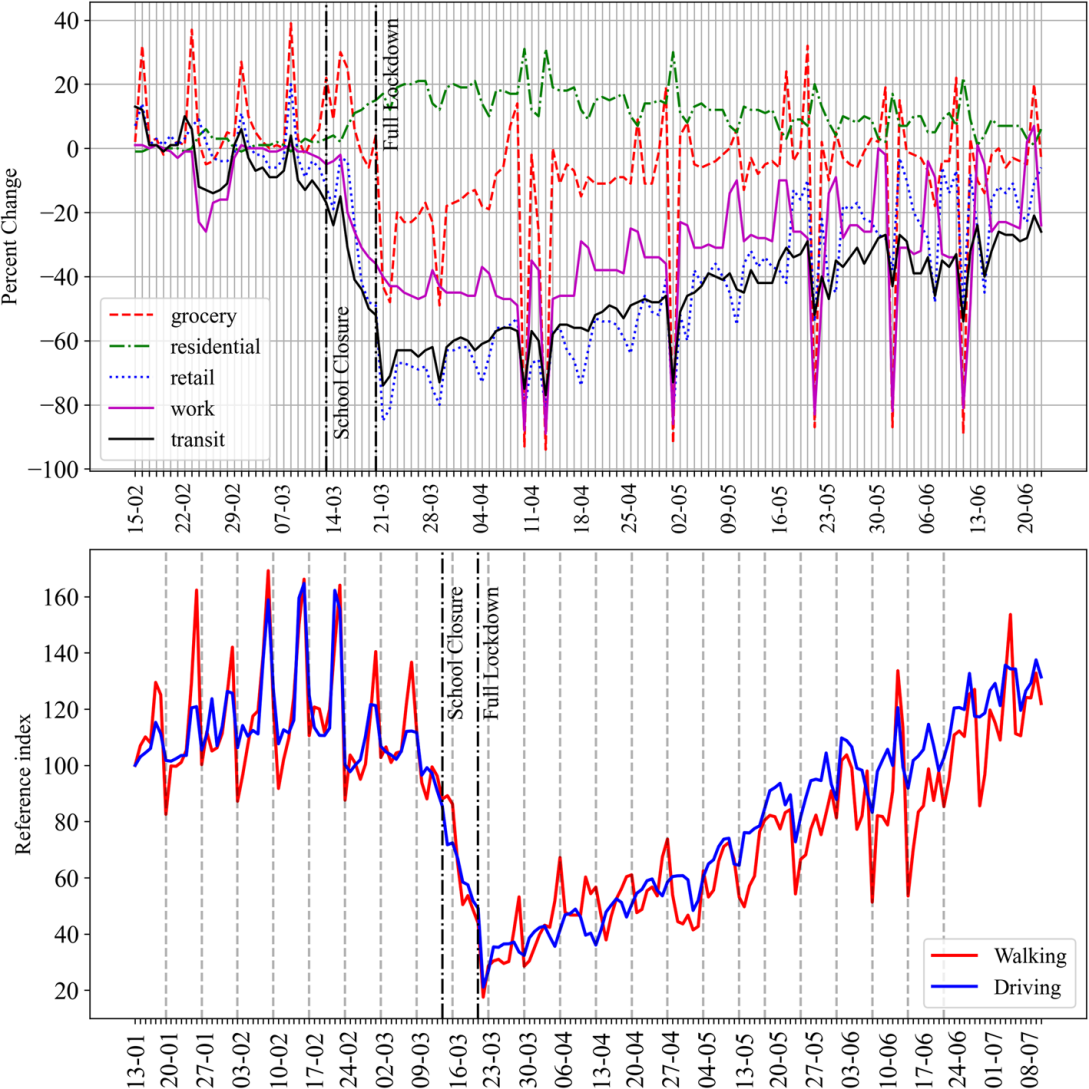
(Top) Activity patterns in Bavaria and (Bottom) travel mode patterns in Munich, data source: [17, 18]

The study of human activity and travel behavior is traditionally (and commonly) based on the data from Stated and Revealed preference surveys. Emerging sources of data [14], such as social media [15] or mobile phones [16], have pushed the use of data-centric approaches to study activity patterns. Alternate data sources can play a crucial role, especially during situations like COVID-19, when responses or policies have to be adopted faster, whereas surveys take some time in planning and execution. This aspect became prominent during COVID-19 when several organizations came forward by making some of their data publicly available to help governments and citizens understand the changes in activity patterns and travel behavior. Some of the prominent examples are COVID-19 Community Mobility Reports [17] and Apple Mobility Reports [18]. For instance, we obtain activity and mobility trends for Munich (in Bavaria, Germany), which provide information about the overall changes in activity and travel mode patterns in a region, respectively (Fig. 1) from [17, 18]. In the figure, the overall activity and mobility trends confirm some expected behavioral patterns during COVID-19 such as a decline in transit mode use, drop in retail and workplace-related visits, and increase in stays at residences. The grocery related visits can be seen to gradually recover from the initial drop in visits.

During COVID-19, Mobile phone data emerged as a potential source to understand and respond to the pandemic, as it provides a large spatio-temporal information [19]. A study using the mobile phone data found that the lockdown in France caused a 65% reduction in the performed trips, especially work-related trips during peak hours and long trips [10]. Researchers in the US and China also applied the mobile phone data to establish that the social distancing and decreased mobility (due to restrictions or lockdowns) is positively correlated to the reduced growth in COVID-19 cases [20, 21]. These few examples illustrate the potential of passively collected data during the COVID-19 for the informed policy decisions. Apart from the data source, the level of data aggregation also decides its usability. The aggregated datasets (at the city or county level) do not provide detailed information at finer geographical scales such as the POI level and thus have limited applications. On the other hand, disaggregate datasets (at finer geographical scales) could provide richer information for understanding heterogeneity across POIs and demographics [22]. Thus, disaggregate data allows for analysis at a local level for understanding the mechanisms between activity patterns and environmental factors, e.g., at the level of a shop or a transit stop.

Mobile devices or sensors equipped with wireless communication or internet can help understand when, where, and if people are crowding. Crowdsensed information, for example, consists of large datasets built with the help of a large group of people. In Mobile Crowdsensing, individuals “collectively share data and extract information" with the help of a sensing device (like smartphones) towards a common goal [23], such as identifying spatio-temporal patterns of a phenomenon. Crowdsensed data from mobile phones and social media platforms [24, 25], such as Twitter data, can, for example, help to study highly dynamic and disruptive events [26, 27]. Some of these data are available on the internet and could be exploited to create the first line of defense against this pandemic and to develop policies that can mitigate the pandemic’s impact on transport systems and local businesses.

One such exciting and seemingly potential data set is the crowdsensed check-in rate or busyness at the POIs. The crowdsensed check-in rate is the representation (in absolute or relative terms) of the number of people or customers visiting a specific establishment at a given point of time and thus shows its busyness. These data are primarily collected from smartphone applications, in which the user’s location history is enabled, such as geotagged data or Location-Based Social Networks (LBSNs). Geotagged tweets [27], Foursquare check-ins [28], and popularity trends [29, 30] are some examples of such data that capture the spatial-temporal evolution of the demand and have already shown their utility in previous studies. Specifically, popularity trends have been used, to predict venue popularity [30], calculate demand expansion factors [31], classify activities [29] and investigate consumer behaviors [32]. These varied applications of POI demand patterns from popularity trends suggest their potential in other unexplored avenues, such as disruptive events. Certain crowdsensed data such as popularity trends provide only relative or normalized values of the demand for certain activities in certain locations [29]. This is a limitation because it prevents one from inferring the corresponding number of absolute check-ins for which the data is recorded, and thus should be considered when designing methodologies that leverage this information as well as when processing the results. More details about how this limitation is addressed in this study will be provided in the next section, where we introduce both the data and the methodology for this study.

This paper shows that crowdsensed information could also provide some useful insights into the spatial-temporal distribution of the activities or demand during the pandemic situation. Subsequently, we propose a model that breaks down POI demand patterns into a set of crucial spatial and other attributes, which are assumed to explain the POI demand. This paper is structured as follows: Section 2 presents the study’s methodology; Sections 3 and 4 concern data collection and data analysis respectively, Section 5 presents the results. Section 6 discusses the findings and limitations, and lastly, we provide a set of conclusions from the research.

## Methodology

We use POI visitation data (response variable, denoted by *P*) and check their correlation with the spatial and other attributes (explanatory variables) of the POI. Firstly, we define a bounding box for the study area and identify the POIs within that area. For these identified POIs, we collect the historical popularity data and live popularity data on different days to capture the before-lockdown and during-lockdown situation, respectively. Some of the prominent spatial attributes that could affect customer visits at a POI are population density [33], parking facilities [34] and public transit stops [33] nearby the POI. As we aim to capture the spatial variability among the POIs, the selected spatial attributes should capture the local variation, e.g., threshold distance for calculating population around a POI should not be too large. Because of this, a square bounding-box of side 600 m (two times the assumed neighborhood distance of 300 m) is used to calculate the population living within the catchment of a POI. Similarly, for the parking area, the catchment corresponds to a square bounding-box of side 50 m. Here the distance threshold is selected to characterize short walking distance because parking far from the supermarket is discouraging for the customers [34]. We adopt the same threshold for all POI types, but in doing so, we ignore the effect of the POI type on the catchment distance, as it is treated uniformly for all POIs. To compute the average distance between a POI and transit stops, we identify the stops within a straight-line distance (“as the crow flies”) 400 m of a POI. This selection of straight-line distance threshold could even result in walking distances greater than 400 m in some cases because the actual route length may be longer depending on the street network. Commonly transit agencies use a walking distance of 400 m as a thumb rule for measuring neighborhood accessibility and transit accessibility, as reflected in previous studies in accessibility research [35, 36]. However, it is relevant to point out that the walking behavior is determined by a multitude of factors such as trip purpose, built-up environment, mode type, and population demographics [37, 38], and thus a detailed sensitivity analysis is beyond the scope of this paper. Average transit stop distance is the average distance of all the identified stops from a POI. Finally, weather features specific features such as temperature and precipitation can also be relevant for studying demand [39].

Non-spatial attributes are the POI type (e.g., supermarket or chemist); the number of reviews and ratings of a POI, e.g., the supermarket’s temporal demand pattern, could be different from that of a fast-food outlet shown in [29]. Further, if a POI has a high positive rating, it could imply its high likeability or customer satisfaction. Similarly, a large number of reviews by customers could be indicative of latent characteristics of a POI. These features such as rating and number of posted reviews are also used in modeling the demand trend modeling [30, 32]. It is pertinent to mention that other demographic factors, such as average income in the locality, could also play an essential role in the retail consumption [33], but the same is not considered in this study because such a dataset was not available.

### Data sources

POI data is collected from Open Street Maps (OSM) via Overpass-Turbo [40]. Google’s Popular time graph data [41] is collected as a measure of the demand patterns at all the identified POIs. A popular time graph shows the busyness (workload or saturation) of a POI during the day, relative to the busiest time during the week [41]. Historical busyness is quantified on a relative scale of [0,100], where 100 indicates the busiest hour. This information is derived from the anonymized and aggregated data from the POI visitor’s location history [41]. As per Google, if the number of such users (who have opted for the location service) visiting a POI is not sufficient, then the popular time graph and the place’s live visit data may not be available [41]. For a given POI, a Popular time graph is averaged over the last few months [41], which could be referred to as “historical popular time”. Live visit data shows the popularity in real-time, which in some cases could be greater than 100 depending on its busyness or crowding relative to the past trends. The popular time data for a particular POI is publicly viewable on the Google Maps website [42]. Due to the smartphone-based passive data collection, Popular time data could also suffer from sample bias. As mentioned above, Popular time data is relative information and cannot infer the actual number of visitors without extrinsic information. Based on the above, we argue in this paper that live data could be an important indicator for measuring changes in the demand as, for each POI, they provide a measure of the deviation between the current and the average venue popularity.

Population data are obtained from the publicly available High-Resolution Population Density Maps provided by Facebook [43]. Facebook used state-of-the-art Computer vision techniques to process satellite imagery and generate this data. Population data provide human population distribution at 30-meter spatial resolution. Parking area (size and locations) and transit stop locations are obtained from the OSM data (obtained via Overpass [40]) and GTFS [44], respectively. Python library OSMNX [45] is used for the processing and analyzing OSM data.

### Modeling approach

The study aims to examine the effect of the lockdown restrictions on the popularity of POIs. This is a problem of causal inference framework, where lockdown acts as a treatment variable. With pre-treatment and post-treatment data, a preferred modeling approach based on causal inference framework could be adopted by controlling for the treatment (lockdown) and confounding (day-of-the-week, POI type) variables. Herein we check the significance of covariates in explaining the day-specific popularity before and during the lockdown. Previous studies such as [32] and [30] have also modeled popularity as a dependent variable in regression formulation. The dependent variable is the day-specific popularity of a POI, which is to be mapped to a set of explanatory variables, represented analytically as follows:

1 $$ \begin{aligned} {P}_{i-d} = f(p_{i}, {pa}_{i}, {sd}_{i}, r_{i}, {nr}_{i}, {type}_{i}, L_{d}, D_{d}, T_{d}, {Pr}_{d}) + \epsilon_{i-d} \end{aligned}  $$

Where,

- *P*_*i*−*d*_ is the response variable in terms of popularity of *i*^*th*^ POI on day *d*
- *p*_*i*_ is population within the defined catchment of *i*^*th*^ POI
- *pa*_*i*_ is the total parking area within the defined catchment of *i*^*th*^ POI
- *sd*_*i*_ is the average distance to the transit stops within the defined catchment of *i*^*th*^ POI
- *r*_*i*_ is the rating of *i*^*th*^ POI
- *nr*_*i*_ is total number of reviews of *i*^*th*^ POI
- *type*_*i*_ is the dummy variable of *i*^*th*^ POI type namely, supermarkets, chemists and fast-food
- *L*_*d*_ is the lockdown dummy variable, wherein during lockdown *L*_*d*_=1; for historical data *L*_*d*_=0
- *D*_*d*_ is the dummy variable representing the day of the week e.g., Monday, Tuesday, and so on.
- *T*_*d*_ and *Pr*_*d*_ are weather specific covariates for temperature and precipitation, respectively, on day *d*
- *ε*_*i*−*d*_ is the residual term

POI type (*type*), lockdown (*L*) and day (*D*) are categorical variables, and are used as dummy variables after one-hot encoding e.g., for a supermaket POI, *supermarket* = 1, whereas *fast-food* and *chemist* are assigned 0 values. Similarly during the lockdown, *lockdown* = 1, else *lockdown* = 0; and on a Monday, only *monday* = 1, while other day-of-the-week dummy variables are equal to zero.

Linear regression models are simple and intuitive as they help to understand the average or global effects of the features. However, these models depend heavily on the explicit analytical formulation and thus could introduce model-bias. To counter this, we use regularized Gradient Boosting (GB) [46] for regression, inspired by previous studies [30]. GB, a machine learning technique, is based on training weak learners in an additive manner. Unlike linear models, GB models do not need an analytical specification and are also less sensitive to outliers. GB can work well with small data while avoiding overfitting. The use of regularized objective function in equation 2 helps to control overfitting. We refer to the regularized GB as Gradient Boosting Regression (GBR) model in this paper.

2 $$\begin{array}{@{}rcl@{}} \mathcal{L}^{(t)}=\sum\limits_{i=1}^{n} l\left(y_{i}, \hat{y}_{i}^{(t-1)}+f_{t}\left(\mathbf{x}_{i}\right)\right)+\Omega\left(f_{t}\right) \end{array} $$

where **x**_*i*_ denotes the *i-th* instance of the dataset of size *n*; *f*_*t*_ is the current model fit; *l* is the loss function which measures the difference between the target (*y*_*i*_) and the prediction $\hat {y}_{i}$, at *t-th* iteration; *Ω*(*f*) is the regularization term to check over-fitting. The details of the GBR are given in [47] and [46].

The best GBR model is selected based on the lowest Mean Squared Error (Eq. 3) on the training data (90% split), using 10-fold cross-validation. The main tunable parameters for the GBR model are the number of estimators and the tree depth [48]. To handle overfitting, we check the MSE on the test data (10%) to ensure that training and test errors are close.

3 $$\begin{array}{@{}rcl@{}} \text{MSE}=\frac{1}{n}\sum_{i=1}^{n}\left(y_{i}-\hat{y}_{i}\right)^{2} \end{array} $$

The interpretation of tree-based models, like the GBR model, is not straightforward since single coefficients (as in linear regression models) for attributes are not available. There are many tools for global interpretation, i.e., the average impact of the features on the model output. Recently, work has been done on the local explanations of these models to uncover the role of each feature for every model instance. The combined behavior of these local explanations can also infer the global behavior. In this regard, SHAP (SHapley Additive exPlanations) is a recently developed Python tool for explaining a machine learning model’s outputs using the game-theoretic approach [49]. TreeExplainer method from SHAP, calculates classic Shapley values (a concept from the game theory [50]) and assigns importance or credit to the input features based on their role in the particular model prediction [51]. Similarly, local interaction effects are captured based on the Shapley interaction index from game theory by allocating the credit to a pair of features [49]. A novel advantage of TreeExplainer is that it can compute Shapley values for tree-based models in polynomial time [49], which makes them highly efficient for practical applications. For details on SHAP, we refer the reader to [49, 51].

We use Ordinary Least Squares (OLS) regression (linear regression), as a reference model for checking the consistency in the interpretation of the global effect of the features (Eq. 4). It can be seen that, in addition to the main effects, we also include interaction effects of lockdown (*L*_*d*_) with all the other variables. It is pointed out that in the linear model, the coefficient (*β*_8_) of *lockdown* gives the effect of lockdown on the *chemist* POIs, conditional on the other covariates. Thus, the coefficient (*β*_8_) actually represents interaction effect of *lockdown-chemist*. We do not include *chemist* dummy explicitly in Eq. 4, as it is highly negatively correlated with *supermarket*.

4 $$\begin{array}{@{}rcl@{}} P_{i-d} &= &\beta_{0} + \beta_{1} \cdot p_{i} + \beta_{2} \cdot {pa}_{i}+ \beta_{3} \cdot {sd}_{i} + \beta_{4} \cdot r_{i} \\&&+ \beta_{5} \cdot {nr}_{i} + \beta_{6} \cdot supermarket_{i} + \beta_{7} \cdot {fast{-}food}_{i} \\ &&+ \beta_{8} \cdot L_{d} + \beta_{9} \cdot Monday_{d} + \beta_{10} \cdot T_{d}+ \beta_{11} \cdot {Pr}_{d} \\ &&+ \beta_{12} \cdot L_{d} \cdot p_{i} + \beta_{13} \cdot L_{d} \cdot {pa}_{i}+ \beta_{14} \cdot L_{d} \cdot{sd}_{i} \\ &&+ \beta_{15} \cdot L_{d} \cdot r_{i} + \beta_{16} \cdot L_{d} \cdot{nr}_{i} \\ &&+ \beta_{17} \cdot L_{d} \cdot supermarket_{i}+ \beta_{18} \cdot L_{d} \cdot {fast{-}food}_{i} \\ &&+ \beta_{19} \cdot L_{d} \cdot Monday_{d} + \beta_{20} \cdot L_{d} \cdot T_{d}\\&&+ \beta_{21} \cdot L_{d} \cdot {Pr}_{d} + \epsilon_{i-d}  \end{array} $$

We also use the Robust regression or Robust Linear Model (RLM or M-Estimation) with Huber objective function [52]. This objective function uses two different formulations: least squares (in the center) and least absolute values (in the tails), basically underweighting the high influence observations or outliers in the dependent variable. Finally, it is noteworthy to refer to a recently published study that also uses both the GBR and linear regression for modeling and SHAP to explain building energy performance [53], due to inherent similarity in our modeling approaches. Modeling is done using Python programming language using the following libraries: statsmodels [54] and XGBoost [48].

## Data collection and processing

We select Munich (the Free State of Bavaria’s capital city in Germany) as the study area. Even though many countries in the world are affected by COVID-19, the extent of the impact depends on multiple factors, such as first COVID-19 incidence [55], rate of spread, travel restrictions [56], testing and contact tracing & containment [57], amongst many others. Therefore, the data for before-during scenarios were collected based on the restriction or lockdown timeline. In Germany, the need for social distancing was announced on 12.03.2020, followed by the announcement of the temporary closure of schools on 14.03.2020 and non-essential travel ban on 18.03.2020 [58]. The Federal States took up state-specific measures depending on their needs, as the imposition of a full lockdown in Bavaria on 20.03.2020 [58]. Therefore, it can be concluded that the second and third weeks of March were the transition period from pre-lockdown to the lockdown period. We are also interested in exploring how the demand pattern at a POI evolves during different stages of the lockdown (e.g., during the early lockdown in the third week of March viz-a-viz during the late lockdown in the last week of April).

**Fig. 2:**
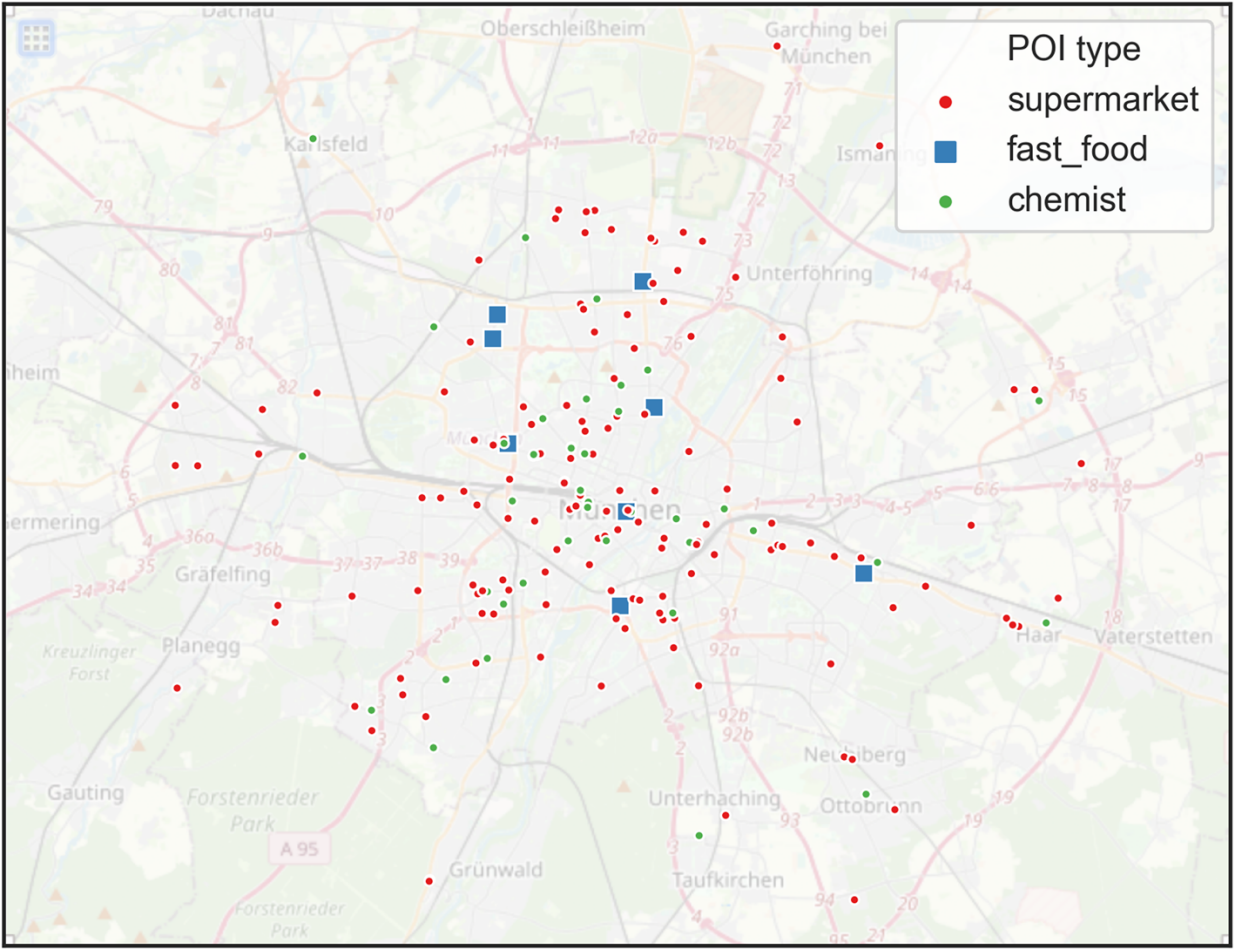
Spatial distribution of POIs with Live data^1^ Basemap source: Open Street Maps

3283 number of POIs were initially identified in the bounding box around Munich [*latitude*:48.137585±0.1125,*longitude*:11.575444±0.175]. The POI attributes, namely location (latitude and longitude), type, rating (on a scale of 1-5), and the number of reviews, are collected. For these POIs, we use python library [59] to obtain hourly historical data (Table 1). The live data is collected bi-hourly, e.g., 1200 H, 1400 H, 1600 H, etc. Not all of these POIs are found to have live popularity information during the lockdown, possibly due to the temporary closure of such POIs due to restrictions or insufficient users visiting such POIs. The availability of the live data varies per hour-day. Therefore, for the subsequent analysis and modeling, POIs without live data during 1200-1800 hours are dropped. The analysis period of 1200-1800 is chosen to represent the consistent working time for all the POIs, away from the opening (around 0800-1000 H) and closing hours (1900-2000 H). Only POI types with at least five samples are selected to ensure representativeness, which leaves a total of about 180 POIs for three categories (Fig. 2), namely supermarket, fast-food, and chemist (Table 2). The low number of POIs makes sense because several retail and leisure POIs, such as restaurants, stores, and barber shops, were closed and severely affected due to the lockdown restrictions, and that is why we suppose no popular time data were available for such POIs.

**Table 1:** Popular time data collection

Date	Cumulative	Type of	Description
	COVID-19 Cases ^∗^	data (Period)	
13-02-2020	−	Historical average (0000-2400)	Before lockdown
20.03.2020	878	Live (1200-1400)	Start of lockdown
03.04.2020	3304	Live (1200-1400)	Middle of lockdown
14.04.2020	4714	Live (0800-2000)	Late lockdown
22.04.2020	5332	Live (0800-2000)	Late lockdown
27.04.2020	5607	Live (0900-2100)	Late lockdown

^∗^ source: [63]

**Table 2:** Number of identified POIs with historical data and live data

POI type	With historical data	With live data ^∗^
Supermarkets	262	137
Fast-food	170	8
Chemist	73	35

^∗^live data availability varies per day

## Data analysis

**Fig. 3:**
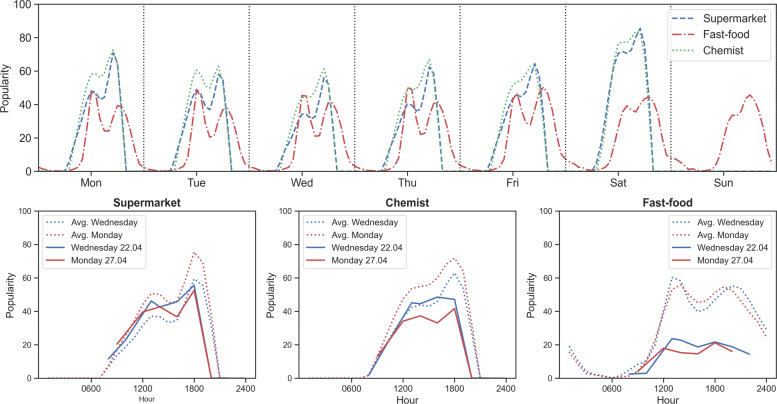
(Top) Historical average popular time trend and (Bottom) Live popular time trend for the three POI types

The hourly trends of average historical popularity in three types of POIs, namely supermarkets, chemists, and fast-foods, are shown in Fig. 3. In the historical trend, supermarkets show a prominent peak during the evening hours, coinciding with the evening commute. Chemists also show a similar pattern. The trend is absent on Sunday, as most supermarkets and chemists are closed on Sundays in Munich. The fast-food category trend shows two prominent peaks during the weekdays, which can be attributed to busyness during lunch and late-evening hours. The demand trend on the weekend shows a high demand from lunch to late-evening hours.

**Fig. 4:**
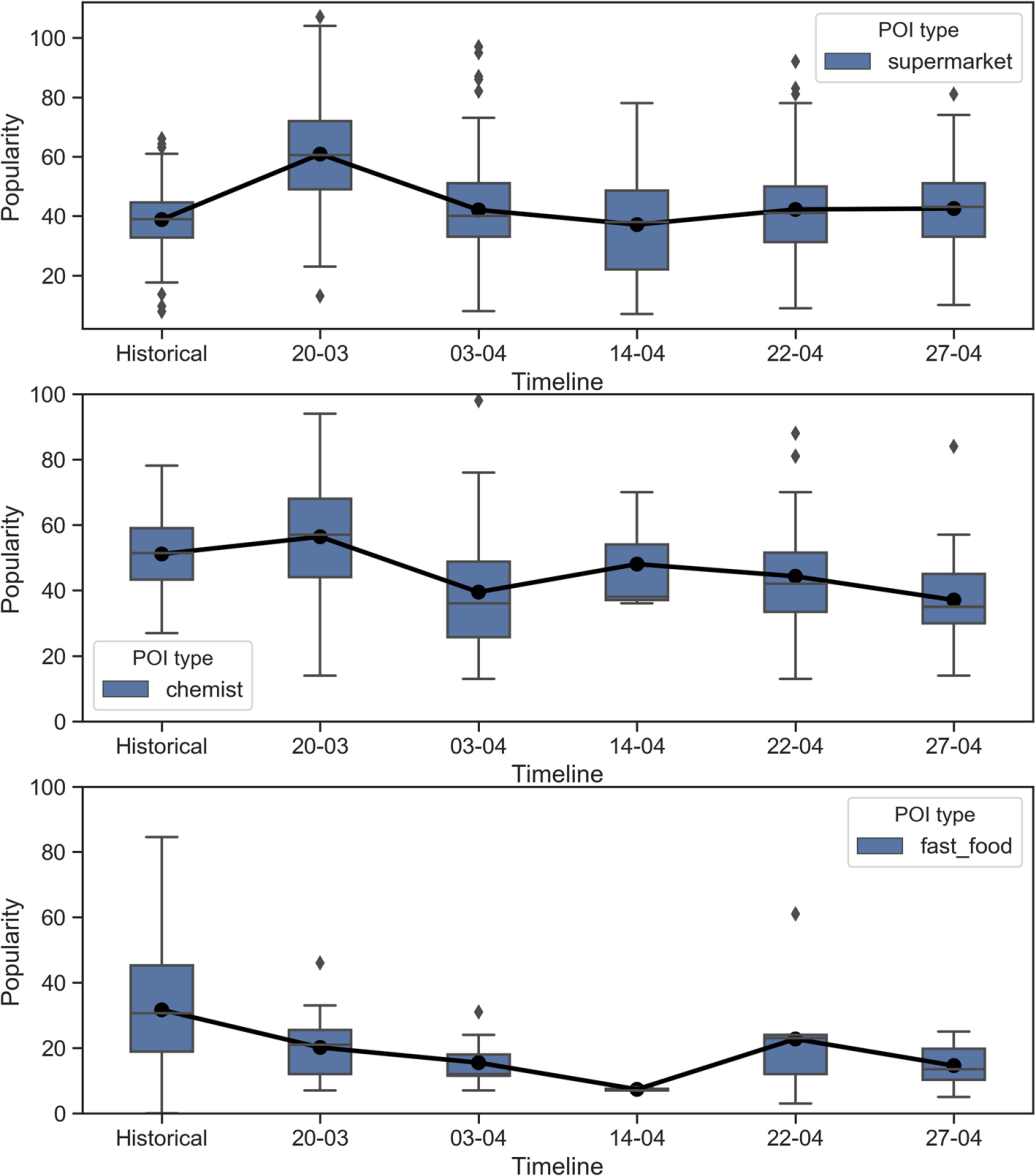
Live popular time trend at 1400 H on different days during the lockdown

Figure 3 shows the average live trends on two days of the week during the lockdown, 22nd April (Wednesday) and 27th April (Monday). Compared to the average historical popularity, the drop in the peak popularity and the general trend is evident. Interestingly, the drop in the fast-food category is more significant and characterizes lockdown’s adverse effect on similar POIs. It can also be recognized that the shape of the historical popularity trend is different on Monday and Wednesday for both supermarkets and chemists, indicating variations during the week. The trend of the afternoon (1400 H) popularity on few selected weekdays also shows the effect of lockdown on the three POI categories (Fig. 4). Again, supermarkets and chemists show similar trends with average historical popularity at around 40-50% of maximum popularity, but markedly increasing on 20th March, i.e., the day lockdown was announced. This increase (57% for supermarkets and 10% for chemists) could result from panic buying for groceries and health retail because of the uncertainty in the early days of the lockdown and pandemic. During the later lockdown period in April, a gradual recovery of the popularity of the supermarkets and the chemists’ POIs is observed. The fast-food category trend is distinct by fall in its popularity, which did not wholly recover in April, although it shows small signs of recovery. It can also be seen that there is no panic buying in the fast-food category on the day of the lockdown announcement, unlike the other two types of POIs.

The summary of the explanatory variables is given in Table 3. The parking area locations in OSM correspond to a mix to different parking types such as surface parking, multi-level parking and underground parking. The composition of the parking areas in our sample is surface (70%), underground (3%), multi-storey (1%) and missing label (26%). We use the historical data, and live data (on 22.04.2020 and 27.04.2020) for modeling pre-lockdown and during lockdown scenarios, respectively (Table 1). The response variable in the regression models is the average of the popularity at two-hour interval over the period of 1200-1800 H, as follows:

5 $$ \begin{aligned} {P}_{i-d} = \left(P^{1200}_{i-d} + P^{1400}_{i-d} + P^{1600}_{i-d} + P^{1800}_{i-d}\right)/4 \end{aligned}   $$

**Table 3:** Summary of the explanatory variables

	Population	Parking area	Transit stops	Avg. Stop	Rating	Reviews
	(<300 m)	*m*^2^ (<50 m)	(<400 m)	distance (m)	(1−5)	
minimum	195	0	1	105.2	2.5	8
mean	2614	414.3	8	324.8	3.9	371
maximum	4340	4503.7	28	794.0	4.9	4742
*σ*	727	850.1	5	87.4	0.3	551

where, $P^{t}_{i-d}$ is the popularity at time t

The features such as rating and number of reviews change with time as new users rate and review a specific POI. In our case, the change is found to be marginal, i.e., the mean percentage change in rating and reviews during the analysis period is found to be 0.2% and 0.0%, respectively. We do not control the weather-specific covariates due to the panel’s limited dimension (two days of live data). The weather for these two days was similar as characterized by sunny or partial cloudy [60], which makes it reasonable to not control for weather-specific covariates. In the case of sufficient panel data, we recommend controlling for weather covariates for precise model estimation.

## Results

**Fig. 5:**
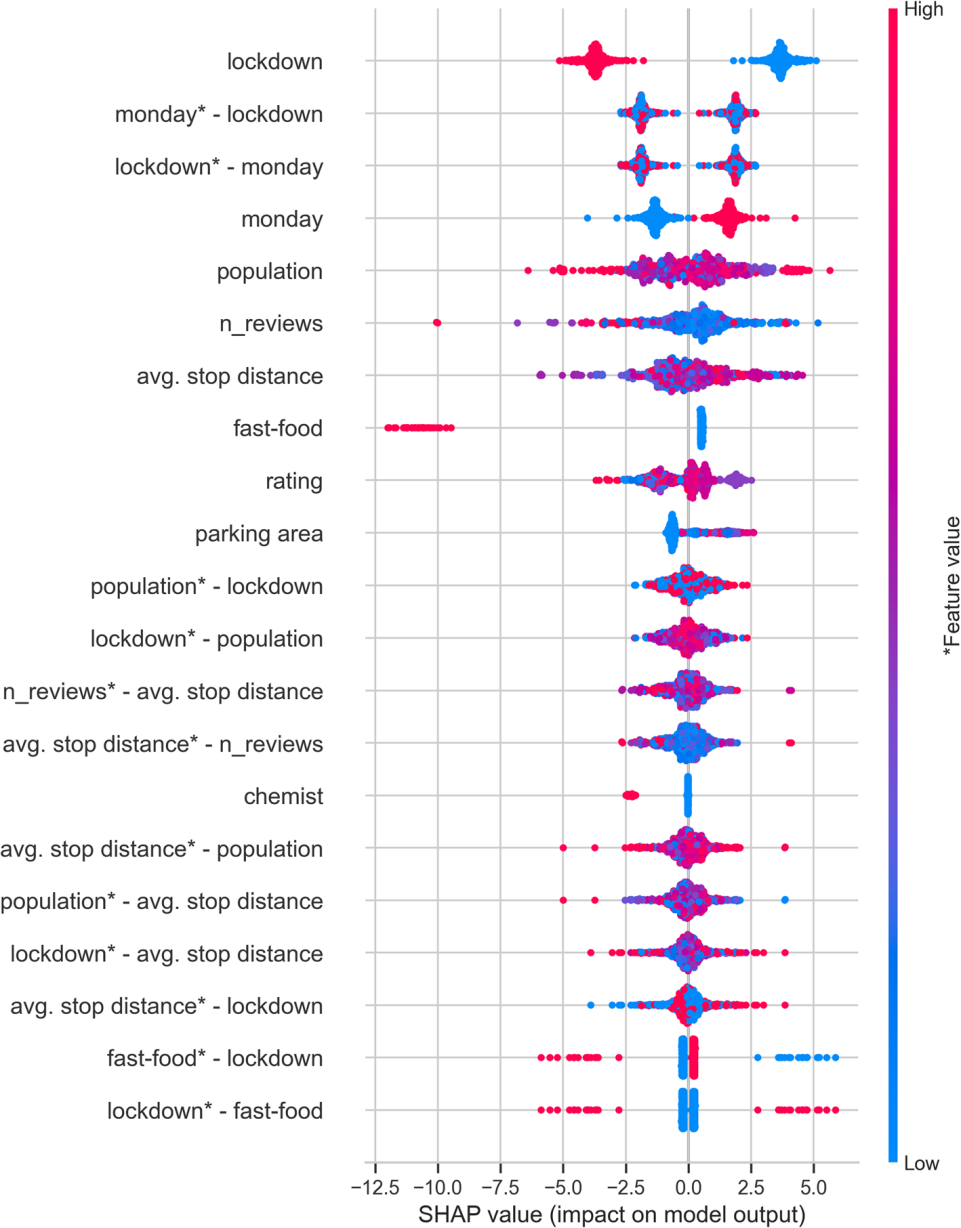
Feature impact based on SHAP values for the 15 largest main and interaction effects [49]

Using cross-validation, we identify the best parameters for the GBR model (number of estimators: 20, maximum tree depth: 4). With these parameters, the model achieves an *R*^2^ of 0.63. The MSEs obtained on the training (7.4), and test data (9.6) are close, which implies no over-fitting. In the SHAP summary plot (Fig. 5), the feature impact on the output of the GBR model is shown with the distribution of SHAP values. In these plots, each point corresponds to one POI instance in the dataset and corresponding SHAP values of the features. The color represents the feature value (blue for low value and pink for high value). The features in these sub-plots are ordered by the sum of the SHAP values’ magnitude over the training dataset. If high SHAP values are observed for corresponding high values of the feature, it means an increase in that particular feature results in an increase in popularity and vice-versa. If SHAP values for a feature are concentrated near 0, that particular feature does not play much importance in predicting the popularity.

The main effects of *lockdown*, *monday* and *fast-food* are clear due to distinct distribution SHAP values for low and high feature values. The *lockdown* feature is found to be correlated with the drop in popularity. The popularity on Monday is found to be higher than that on Wednesday. It is interesting to note that the POI type plays an important role, especially for fast-foods. The *fast-food* attribute is found to be correlated with low SHAP values (i.e., *fast-food* =1), which pushes the popularity to the lower side. The impact of the *population* and the number of reviews (*rating_n_x*) is not clearly correlated with the popularity value, as evident by overlapping pink and blue points. The low values of the *parking area* feature show low SHAP values, whereas high *parking area* is associated with high SHAP values (albeit with some overlap); i.e., it pushes the POI popularity to the higher side. Similarly, the type of POI, namely *chemist*, is correlated with the decrease in popularity as evident from negative SHAP values. Hence, the features viz. *lockdown*, *day-of-the-week*, *POI-type*, and *Parking area* show a clear correlation with popularity.

**Fig. 6:**
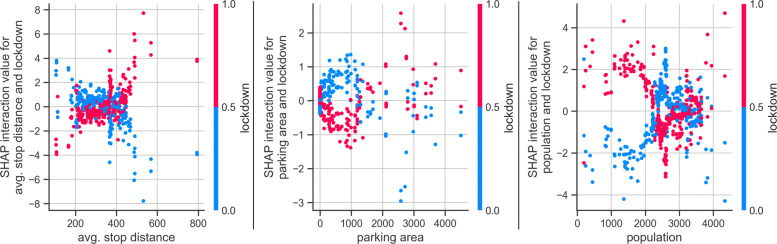
SHAP dependence plot based on local explanations for the spatial features’ interaction with the lockdown

Figure 5 also shows the interaction effects, where the superscript ∗ indicates which feature is represented by the color bar. The interaction effects of *lockdown* and *fast-food* features also show clear effects, implying the adverse effect of lockdown on the fast-food POIs in terms of popularity, also seen in Fig. 3. Spatial factors, *population*, and *avg. stop distance* are found to have mixed effects (overlap of pink and blue points), and thus their global effects on popularity are not clear in Fig. 5. However, the interaction effect of *lockdown - avg. stop distance* (see feature *avg. stop distance* ^∗^- *lockdown*) shows high SHAP values for some longer stop distances, and vice-versa. This effectively means that POIs close to the transit stops had lower popularity than those farther from a stop during a lockdown. This is even more clear in the local explanation plot in Fig. 6, wherein the interaction effects of *lockdown - avg. stop distance* are inverted during the lockdown.

The fit of the OLS and RLM models is not as good as that of the GBR model, as evidenced from lower Adjusted *R*^2^ values (Table 4), which also justifies the use of the GBR model as it introduces less bias as compared to the linear models. Nevertheless, the results of the OLS model (the sign and magnitude of the coefficients) show that the average behavior of the features is consistent with that of the GBR models. In both the OLS and RLM model, the intercept term is found to be significant, with a value close to 50. The main effect of *lockdown* is not found to be significant, unlike in Fig. 5. In the linear model (Eq. 4), it represents interaction of *lockdown-chemist*. The main effects of only *monday* and *average stop distance* are found to be significant. The positive coefficient of *monday* shows that the average historical popularity of POIs on Monday is more than that on a Wednesday due to variations in the daily demand patterns (Fig. 3). The negative coefficient of the *average stop distance* implies that popularity decreases with an increase in the distance to a transit stop. The *lockdown* has significant interactions with other features. The popularity during lockdown depends on the type of POI, as the interaction of *fast-food* type POI has a greater negative coefficient than the other two types of POIs, whereas the popularity of the supermarkets is marginally greater than the chemist type POIs. During the lockdown, Monday’s popularity is lower than on Wednesday, which provides evidence of the daily temporal variations in popularity during the lockdown. An interesting observation is that popularity is positively correlated with the *lockdown - avg. stop distance* interaction. A possible explanation is a drop in transit ridership during the lockdown, which can be seen in Fig. 1. Specifically, passenger ridership dropped by around 70% during April 2020 in Munich [61], and thus POIs closed to transit stops observed a greater reduction in popularity than others located far from the transit stops. The *lockdown-population* interaction also has a negative coefficient, albeit with a weak significance. One thing to note is that in the linear models, main and interaction coefficients of *parking area* are not found to be significantly correlated.

**Table 4:** Results of Linear Regression

	*Dependent variable:* *P*_*i*−*d*_	
	1:OLS	2: RLM
Intercept	48.74 ^∗∗∗^	53.37 ^∗∗∗^
	(6.93)	(7.04)
fast-food	−2.56	−3.57
	(3.75)	(3.81)
lockdown	3.66	−3.50
	(9.82)	(9.98)
lockdown:fast-food	−19.47^∗∗∗^	−16.73^∗∗∗^
	(5.31)	(5.39)
lockdown:monday	−16.45^∗∗∗^	−16.24^∗∗∗^
	(1.52)	(1.55)
lockdown:average stop distance/1000	19.49 ^∗∗^	20.19 ^∗∗^
	(8.93)	(9.07)
lockdown:number of reviews/1000	0.13	−0.97
	(1.88)	(1.91)
lockdown:parking area/1000	0.18	0.13
	(0.95)	(0.96)
lockdown:population/1000	−1.93^∗^	−1.93^∗^
	(1.10)	(1.12)
lockdown:rating	−1.68	−0.10
	(2.26)	(2.30)
lockdown:supermarket	4.53 ^∗∗^	4.84 ^∗∗^
	(2.02)	(2.05)
monday	11.20 ^∗∗∗^	11.29 ^∗∗∗^
	(1.08)	(1.09)
average stop distance/1000	−10.52^∗^	−11.40^∗^
	(6.32)	(6.42)
number of reviews/1000	−1.52	−1.41
	(1.33)	(1.35)
parking area/1000	0.67	0.64
	(0.67)	(0.68)
population/1000	1.18	1.20
	(0.78)	(0.79)
rating	−0.40	−1.41
	(1.60)	(1.62)
supermarket	−1.96	−2.12
	(1.43)	(1.45)
Observations	718	718
*R*^2^	0.34	
Adjusted *R*^2^	0.32	
Residual Std. Error	10.18	3.21
F Statistic	21.20 ^∗∗∗^	

*Note:* ^∗^*p*<0.1; ^∗∗^*p*<0.05; ^∗∗∗^*p*<0.01

## Discussion

There is a lack of existing research on the effects of COVID-19 on demand patterns at the POI level because this phenomenon is new and not experienced at the same scale in the last 100 years. Activity patterns uncovered in this study match the expectations of viz-a-viz restrictions during the COVID-19 lockdown in Munich. We explained the effect of features in the GBR regression model using SHAP. The behavior of coefficients is consistent with previous studies to some extent, wherein transit stop connectivity is associated with the demand at retail locations [33, 34]. The *population* is not found significant in our model, which could seem counter-intuitive. However, it should be kept in mind that the response variable (POI popularity) is a relative value instead of the absolute value of demand. Significance of *POI type* (fast-food) during the COVID-19 confirms the dominance of POI type in explaining the lockdown impact, possibly as the lockdown was directed to reduce non-essential retail consumption and crowding. POI types are significant in explaining the dip in the POI’s popularity, as *POI-type* captures latent consumer behavior. It is pointed out that the role of the spatial factor might vary depending on city-specific factors like the effectiveness of the lockdown and fall in transit ridership. The behavior (sign) of coefficients in GBR and linear models is similar for most of the features, with some exception such as *parking area*, which furthers the case for the use of advanced models with explainable tools such as SHAP.

The study is naturally not without its limitations. As stated above, relative popularity or demand fails to capture the population’s effect around the POIs. Adding more features, like land-use type (residential vs. workplace), could improve the results, mainly because during COVID-19, generally, work from home was recommended. We also found that live popularity is not available for most of the POIs during the lockdown, limiting the data for modeling and adding to sampling bias. Sensitivity analysis on the effect of sampling variation and feature threshold could be an interesting topic for the future. We do not account for the marketing strategies, which influence consumers. The marketing decisions could be motivated by a complex set of factors such as weather, time, day, and month, and thus to some extent, the overall effects can be captured by collecting time-series data and controlling for an hour, month, and day. But at an individual POI level, marketing-specific data could be hard to collect as the marketing strategies could be diverse and highly dynamic even across similar POI types. Further research should be done on data to infer latent features such as consumer preferences and socializing behavior during disruptive events.

## Conclusions

This research uses a data-driven approach to analyze the activity and demand patterns at the POIs in Munich. We show that POI check-ins are a potential source of information during dynamic events like COVID-19. The use of POI level data and features helps to understand the underlying interactions of spatial and non-spatial features in detail and identify the spatial variability (if any) and the influencing factors thereof. The results are also of interest to the transport planners and operators as they provide insights on the effect of transport variables such as parking area and transit-stop distance on the POI popularity. We provide empirical evidence of the disproportionate effect of the lockdown restrictions on the POIs in Munich, depending on their distance from a transit stop. Businesses near or in the transit hubs are more vulnerable to these disruptions due to reduced commuters, potential customers. This outcome could be due to reduced travel (home-office) or changed travel behavior (customers avoiding public transport). These insights point to the lack of resilience of transit-near POIs due to excessive dependence on commuting customers. Policymakers can look into or even adapt the transit-oriented development principles to diversify the customers of near-transit POIs. It is again highlighted that this study’s findings might not hold for other cities due to the presence of different city-specific factors.

The use of publicly available data sources increases the transferability of this study to other study areas. A time-series crowdsensed data over a longer duration is suitable for causal inference, to conduct the policy impact evaluation of lockdowns, to evaluate the crowding or busyness, and their correlation with the spread of the pandemic. This data could help measure crowding patterns at the POIs, especially when there is an increased need to reduce mobility and contacts. Such patterns could also be correlated with other factors such as public transport schedules, weather, number of infections, and tests to check their influence on crowding behavior. Post-lockdown, the researchers could use the crowdsensed information to analyze if the POI visitation trend has stabilized and returned to normal levels. After all, a pandemic is a kind of disruptive event, and thus similar data could be the potential to study other planned, unplanned or disruptive events. Such insights could help cities to monitor demand patterns and devise effective responses to such events.

## Data Availability

The popular time data analyzed in this study are not publicly available due to data restrictions. Other datasets analyzed during the current study are publicly available at [43, 44] and [62]. Declarations

## Abbreviations

COVID-19: COrona VIrus disease 2019
GBR: Gradient boosting regression
LBSNs: Location-based social networks
OLS: Ordinary least squares
POI: Point of interest
RLM: Robust linear model
RMSE: Root mean squared error
SHAP: SHapley Additive exPlanations

## Footnotes

1 Fast-food POI’s symbol is enhanced for better visibility
